# Comparing the metabolic pathways of different clinical phases of bipolar disorder through metabolomics studies

**DOI:** 10.3389/fpsyt.2023.1319870

**Published:** 2024-01-08

**Authors:** Qin Guo, Jiao Jia, Xiao Li Sun, Hong Yang, Yan Ren

**Affiliations:** ^1^Shanxi Bethune Hospital, Shanxi Medical University, Taiyuan, Shanxi, China; ^2^Department of Mental Health, Shanxi Bethune Hospital, Taiyuan, China

**Keywords:** 1H nuclear magnetic resonance technology, depressive episodes, mixed episodes, hypomania episodes, guanidine acetic acid, trimethylamine oxide

## Abstract

This study identified the metabolic biomarkers for different clinical phases of bipolar disorder (BD) through metabolomics. BD patients were divided into three groups: patients with BD and depressive episodes (BE, *n* = 59), patients with BD and mania/hypomania episodes (BH, *n* = 16), patients with BD and mixed episodes (BM, *n* = 10), and healthy controls (HC, *n* = 10). Serum from participants was collected for metabolomic sequencing, biomarkers from each group were screened separately by partial least squares analysis, and metabolic pathways connected to the biomarkers were identified. Compared with the controls, 3-D-hydroxyacetic acid and N-acetyl-glycoprotein showed significant differences in the BE, BH, and BM groups. This study suggests that different clinical types of BD share the same metabolic pathways, such as pyruvate, glycolysis/gluconeogenesis, and ketone body metabolisms. In particular, abnormal glycine, serine, and threonine metabolism was specific to BM; β-glucose, glycerol, lipids, lactate, and acetoacetate metabolites were specific to depressive episodes; the guanidine acetic acid metabolites specific to BH; and the acetic and ascorbic acids were metabolites specific to manic and BM. We screened potential biomarkers for different clinical phases of BD, which aids in BD typing and provides a theoretical basis for exploring the molecular mechanisms of BD.

## 1 Introduction

Bipolar disorder (BD) is a lifelong episodic mental disorder with a variable course that manifests as depressive, manic, or mixed episodes (1, 2). According to the World Health Organization's World Mental Health Survey, BD has been ranked as the second most important disorder affecting separation from work (3). BD affects 1% of the global population and is one of the leading causes of disability among young people (4).

To date, the BD diagnosis has only relied on the subjective identification of clinical symptoms, and objective diagnostic methods are still lacking. However, the complexity and variability of the clinical manifestations of BD have led to an extremely high rate of misdiagnosis (5). The medications used to treat various clinical phases, and an incorrect diagnosis can lead to misdiagnosis and increased patient suffering, thereby increasing the risk of suicide (6). To address this, a significant portion of research has explored immunoinflammatory or oxidative stress-related markers, such as pro-inflammatory cytokines, zinc, and Thiobarbituric Acid-Reactive Substances (7). However, no specific biomarker has yet been identified for each phase of the disorder (8). Therefore, there is an urgent need to identify stable and reliable biomarkers for different clinical phases BD is urgently needed to improve the accuracy of BD diagnosis (9).

Studies have shown that alterations in different metabolic pathways have been found in BD, including abnormal energy metabolism (10), lipid alterations (11), and abnormal amino acid (12) and glucose metabolisms (13). However, previous studies have not considered the different clinical phases of BD, and the participants of the previous studies were predominantly patients with depressive episodes or stable phases of BD, with no studies on patients with BD and mixed episodes (BM). Metabolomics can capture metabolic alterations in various disease states, and patients with different disease states may have diverse metabolic phenotypes. Nuclear magnetic resonance is the most studied bioanalytical platform in metabolomic research (14). Metabolic studies based on hydrogen 1 nuclear magnetic resonance (1H-NMR) spectroscopy are highly sensitive, reproducible, and quantitative. Thus, 1H-NMR is a well-established metabolomics method (15). Therefore, we used 1H-NMR metabolomic analysis to identify metabolite changes in the serum of patients with different clinical phases of BD and develop objective diagnostic methods.

## 2 Materials and methods

### 2.1 Sample sources

Eighty-five patients aged 15–65 years were recruited from patients who attended the inpatient and outpatient departments of the psychiatry department of the Shanxi Bethune Hospital between January 2018 and August 2020. This research was approved by the Ethics Committee of Shanxi Bethune Hospital. All participants voluntarily participated in the study and provided informed consent.

Inclusion criteria: patients with BD who were diagnosed according to the Diagnostic and Statistical Manual of Mental Disorders, 5th edition (DSM-V) and further grouped based on the current episode type. Hamilton depression rating scale 24 items (HAMD-24) and young mania rating scale (YMRS) were used for evaluation. Patients with BD who met the criteria for depressive episodes according to the DSM-V were classified into the BD depressive episode (BE) group, with no history of mixed episodes in their clinical records. Patients with BD who met the criteria for manic or hypomanic episodes according to the DSM-V were classified into the BD manic/hypomanic (BH) group, with no history of mixed episodes in their clinical records. BD patients who met the criteria for mixed episodes according to the DSM-V were classified into the BD mixed (BM) group (16). None of the patients had received medication or had not received treatment in the previous month. The exclusion criteria were physical or other mental illnesses or substance abuse. In addition, a group of 10 healthy controls (HC) was recruited by advertisement at the same hospital, none of whom had a DSM-IV axis I disorder.

### 2.2 Collection of serum and clinical information

Blood samples were collected from the participants after fasting 12 h. In addition, clinical information such as age, sex, onset time, total disease duration, HAMD, and YMRS scores were collected from all participants and compared using the chi-square test and analysis of variance (ANOVA) among BD subgroups.

### 2.3 H-NMR mapping and data analysis

In this study, a Carr-Purcell-Meiboom-Gill (CPMG) pulse sequence was performed using a Bruker 600 MHz AVANCE III NMR spectrometer with the following parameter settings: free induction decay (64 K data points), self-shunning relaxation latency (320 ms), and 64 scans. The biomarkers were imported into MetaboAnalyst 5.0 (17) (http://www.metaboanalyst.ca/) to identify the relevant pathways of the biomarkers, which were screened using *P*-values based on pathway enrichment analysis and impact values from pathway topology analysis.

### 2.4 Statistical analyses

The serum score data generated by NMR acquisition were centralized and normalized using the SIMCA-P 14.0 software, and all serum samples were analyzed by 1H NMR metabolic profiling using the supervised partial least squares discriminant analysis (PLS-DA). BE (*n* = 59), BH (*n* = 16), and BM (*n* = 10) patients were compared with the HC to screen the differential metabolites in each group. In addition, the MetaboAnalyst 5.0 was utilized to analyze the pathways associated with the differential metabolites. The SPSS 21.0 software was used to analyze the data. The three groups were individually compared with the control group using a *t*-test to identify and evaluate the markers of differences. A *P*-value < 0.05 was considered statistically significant.

## 3 Results

### 3.1 Participant statistics

Table 1 presents the clinical and demographic characteristics of the study participants. As expected, no significant differences in age, sex, or age at first onset between the BE, BM, BH, and HC groups were found.

**Table 1:** Demographic and clinical characteristics of all participants.

**Variable**		**Group**				**Analysis**		
		**BE (*****N*** = **59)**	**BH (*****N*** = **16)**	**BM (*****N*** = **10)**	**HC (*****N*** = **10)**	**df**	***F*** **/** *x* ^2^	***p***
Age (years)		27.16	26.12	23.24	31.67	66	0.109	0.897
Sex	Male	22	7	8	3	2	4.123	0.119
	Female	37	9	2	7			
Onset age (year)		21.13	23.78	20.27	–	57	0.142	0.109
Duration of illness (months)		37.32	27.12	13.16	–	57	0.125	0.089
The total scores of HAMD-24		31.68	3.23	13.67	–	57	8.240	0.02
YMRS		0.12	13.36	4.57	–	57	4.239	0.01

BE, bipolar disorder with depressive episodes; BH, bipolar disorder with mania/hypomania episodes; BM, bipolar disorder with mixed episodes; HAMD-24, Hamilton rating depression scale; HC, healthy control; YMRS, young mania rating scale.

### 3.2 1H NMR spectral data analysis

**Figure 1:**
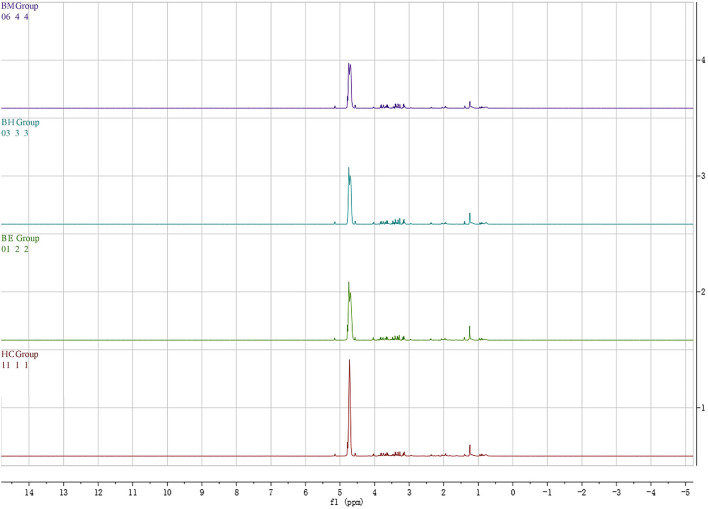
Typical 1H NMR spectrum of plasma in the patients with BD and HC groups. BD, bipolar disorder; BE, bipolar disorder with depressive episodes; BH, bipolar disorder with mania/hypomania episodes; BM, bipolar disorder with mixed episodes; HC, healthy control.

The 1H-NMR metabolite profiles of BE, BM, BH, and HC are shown in Figure 1. Twenty-eight metabolites were identified in the 1H NMR spectra of 95 participants from these groups based on the evaluation of the Human Metabolome Database (HMDB, http://www.hmdb.ca/) and previously published articles (Supplementary Table 1).

### 3.3 Multivariate statistical analysis

**Figure 2:**
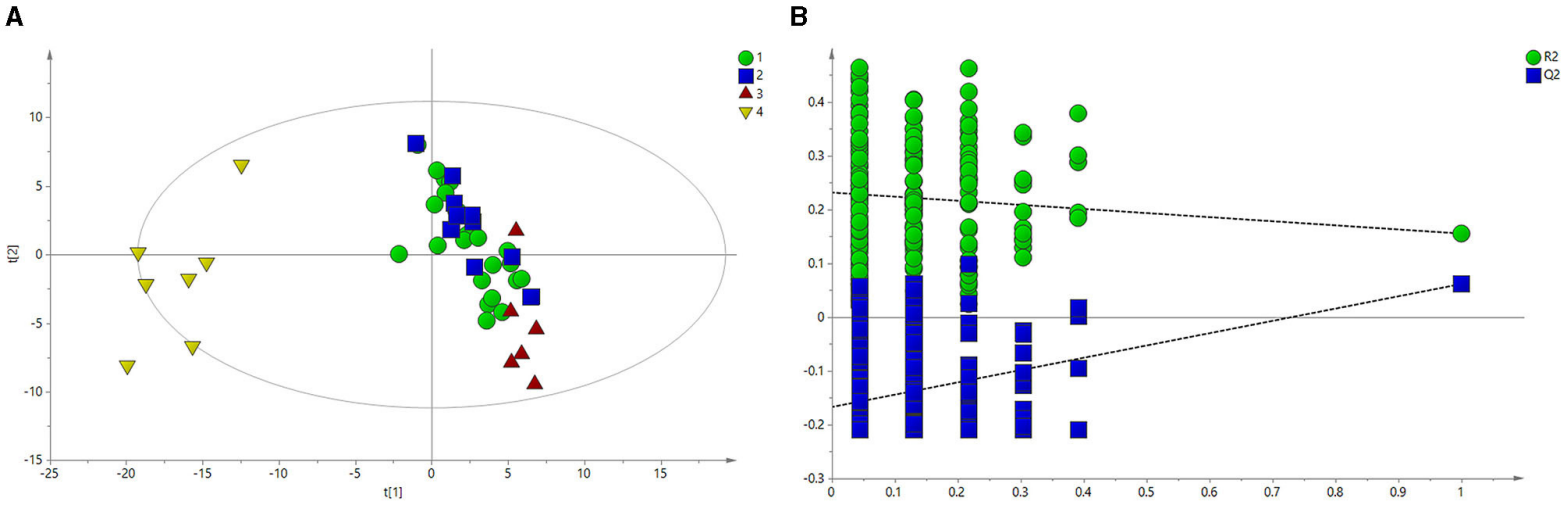
PLS-DA of 1H NMR spectra of serum from BE and HC groups. **(A)** PLS-DA score plots of 1H NMR spectra in which the BE (circle), BH (square), and BM (red triangle) groups were obviously separated from the HC group (yellow triangle). **(B)** 200-iteration permutation test map of the PLS-DA model. BE, bipolar disorder with depressive episodes; BH, bipolar disorder with mania/hypomania episodes; BM, bipolar disorder with mixed episodes; HC, healthy control; PLS-DA, partial least squares discriminant analysis.

Figure 2A shows the PLS-DA results, which revealed that the HC group was clearly separated from the BE, BM, and BH groups. A model validation plot was used to determine whether the PLS-DA model was overfitted. Setting the number of tests to 200, the model validation results were obtained, as shown in Figure 2B. All Q2 values on the left side were less than the original points on the right side, or the regression line of Q2 intersects the vertical axis with values less than zero, proving that the PLS-DA model was valid. Based on the analysis of the macroscopic profiles of the sera of each group, the HC group was clearly separated from the BE, BM, and BH groups, indicating that the BE, BM, and BH groups had changes in their endogenous metabolites compared with the HC group and that the BE, BM, and BH groups were not clearly separated. The above results did not reveal the specific regulation of endogenous metabolites in different clinical phases; therefore, a pairwise comparison of the data of each group is necessary to identify the differential metabolites and analyze the trend of their changes.

### 3.4 Biomarkers analysis

**Figure 3:**
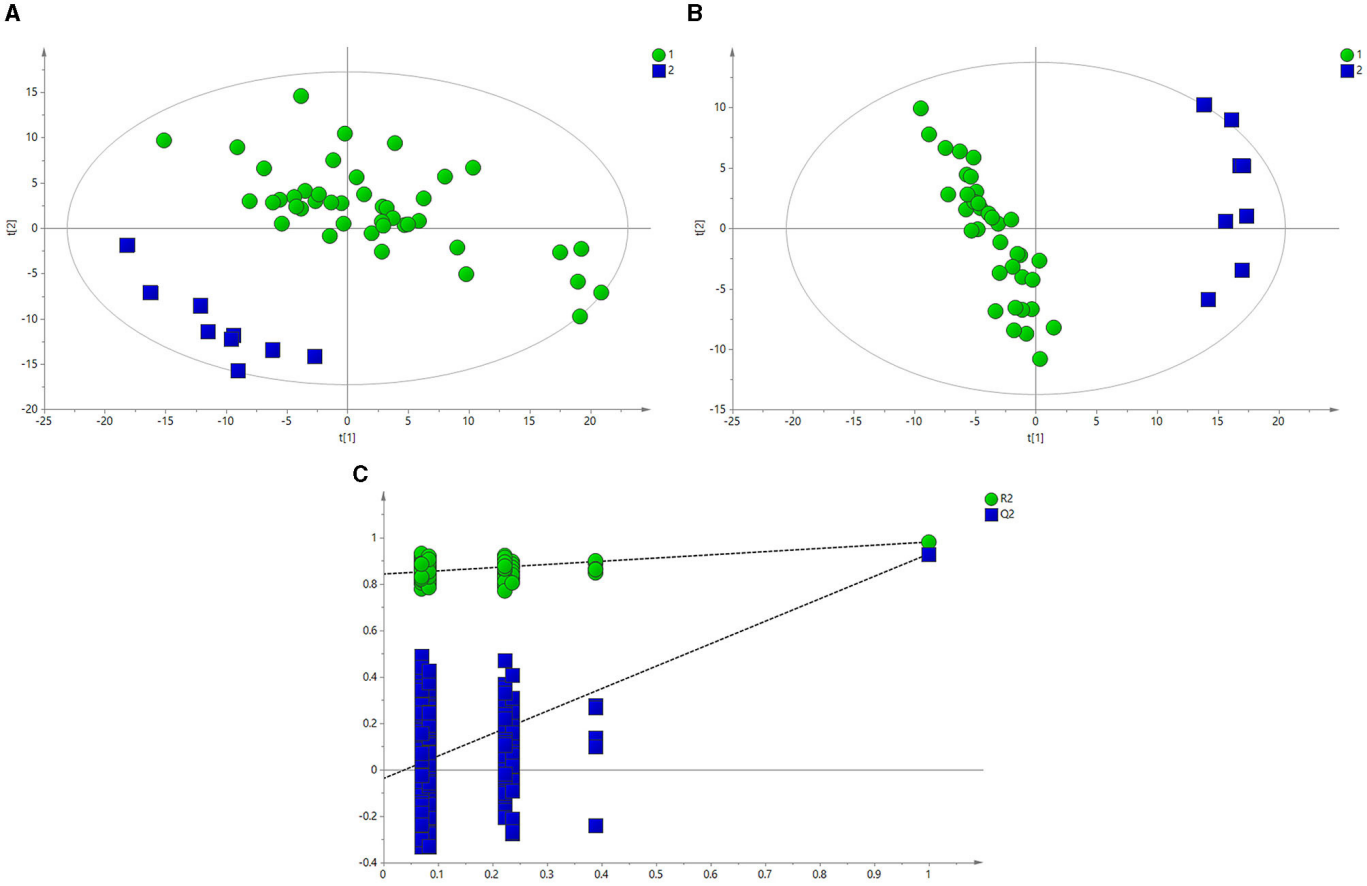
**(A)** PCA score plots of BE and HC groups. **(B)** PLS-DA score plots of BE and HC groups. **(C)** PLS-DA model between BE (circle) and HC groups (square). BE, bipolar disorder with depressive episodes; HC, healthy control; PCA, principal component analysis.

**Figure 4:**
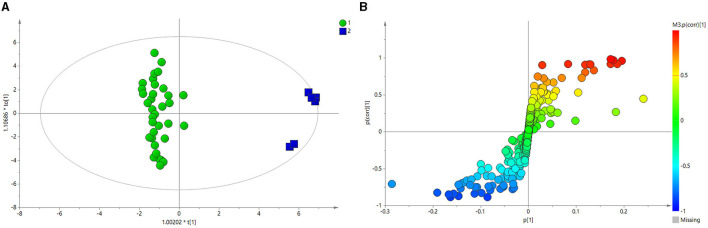
**(A)** OPLS-DA score plots of BE and HC groups. **(B)** PCA plots of the OPLS-DA of BE and HC groups, validated using S-plots. BE, bipolar disorder with depressive episodes; HC, healthy control; OPLS-DA, orthogonal projections to latent structures discriminant analysis.

Figure 3A depicts the principal component analysis (PCA) of the HC and BE groups in response to raw data discrepancies between the two groups. The HC and BE groups were clearly separated, indicating a successful model replication. To reduce within-group error, PLS-DA profile analysis was performed on the HC and BE groups, as shown in Figure 3B. The HC and BE were significantly separated, and 200 model validations were performed, demonstrating the validity of the model (Figure 3C). To reduce within-group target-independent random errors and identify differential metabolites between the HC and BE groups, orthogonal projections to latent structures discriminant analysis (OPLS-DA) was performed, as shown in Figure 4A, to detect differential metabolites with variable importance in projection (VIP) > 1 based on S-plots, and to screen for metabolites with significant differences (*P* < 0.05, *P* < 0.01) by performing independent sample *t*-tests on their peak areas (Figure 4B). Finally, the analysis was outperformed separately for the HC and BM groups and the HC and BH groups, according to the method described above (Supplementary Figures 1A–C, 2A, B, 3A–C, 4A, B).

### 3.5 Changes in potential biomarkers in serum and identification of related pathways

**Figure 5:**
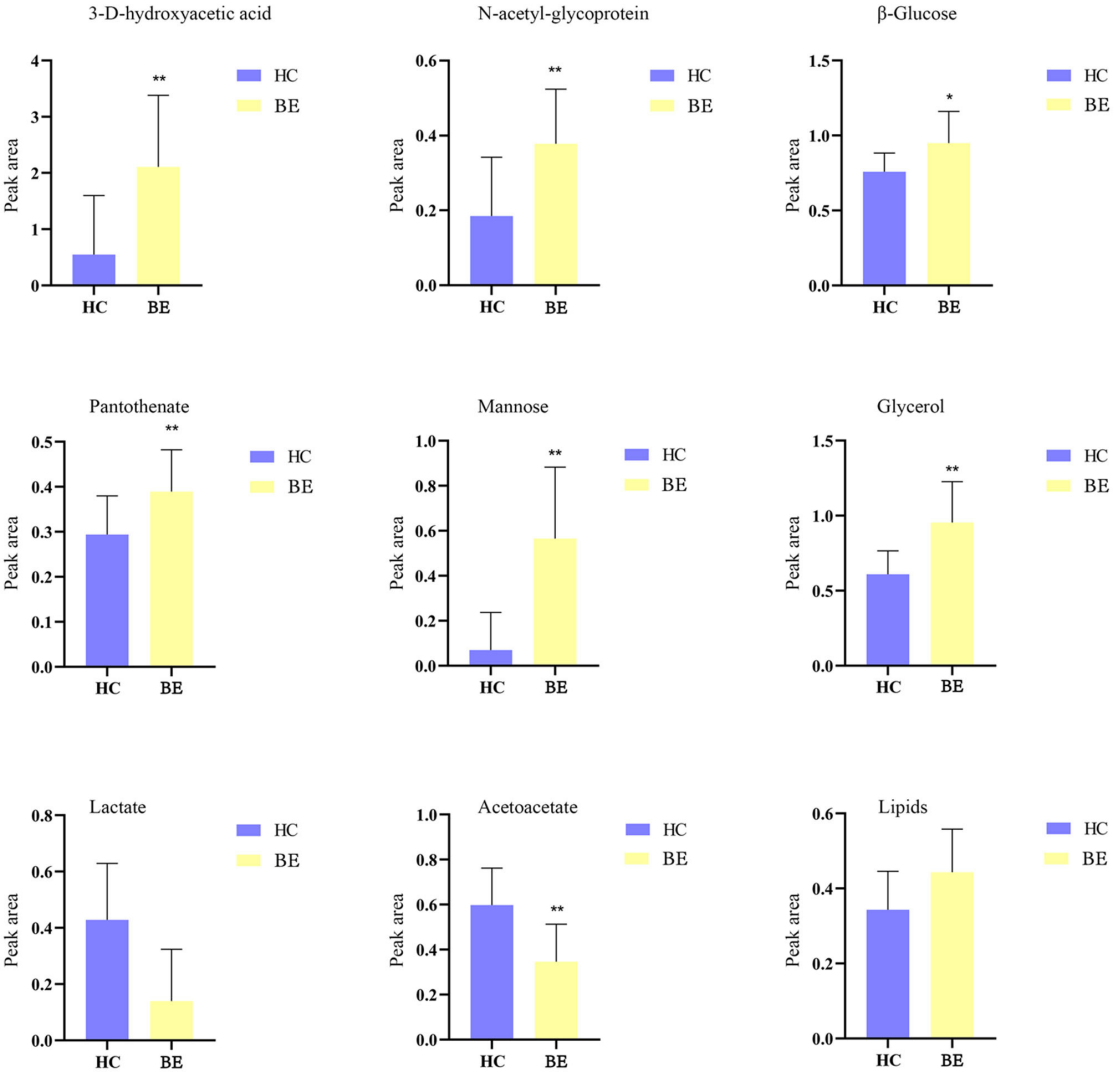
Comparison of relative peak area of potential biomarkers found in serum of BE and HC groups. *P < 0.05, **P < 0.01. BE, bipolar disorder with depressive episodes; HC, healthy control.

**Figure 6:**
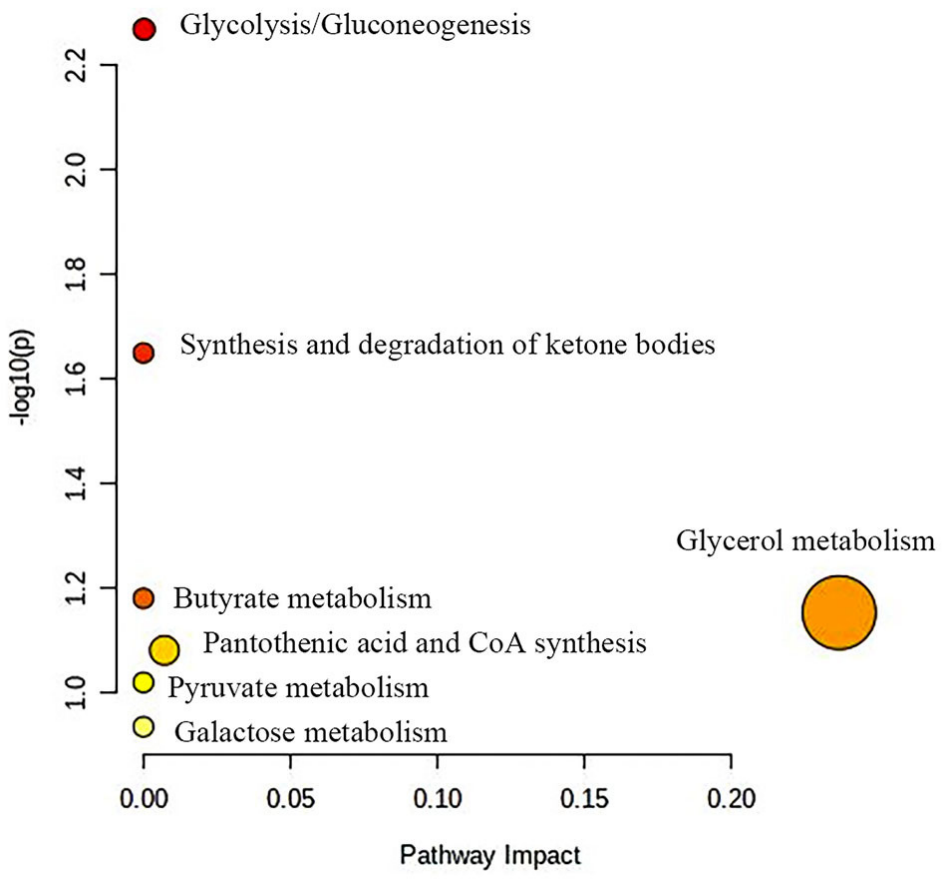
Metabolic pathway analysis of differential metabolites in BE group. BE, bipolar disorder with depressive episodes.

Figure 5 shows the effects of the potential biomarkers in the serum of patients with BE (Supplementary Table 2). Compared with the HC group, seven endogenous differential metabolites were found in the BE group when VIP > 1 and *P* < 0.05. Among them, those with significantly higher levels in the BE group compared with the HC group were 3-D-hydroxybutyric acid, *N*-acetyl-glycoprotein, pantothenic acid, mannose, glycerol, acetoacetate, and lipids (*P* < 0.01). In addition, β-glucose was also significantly higher (*P* < 0.01), and those with significantly lower levels included acetoacetate (*P* < 0.01). MetaboAnalyst 5.0 was utilized to analyze the metabolic pathways in the BE group, and the results are shown in Figure 6. The differential metabolites in the BE group were mainly related to pyruvate metabolism, glycolysis/gluconeogenesis, glycerol metabolism, and ketone body synthesis and degradation.

**Figure 7:**
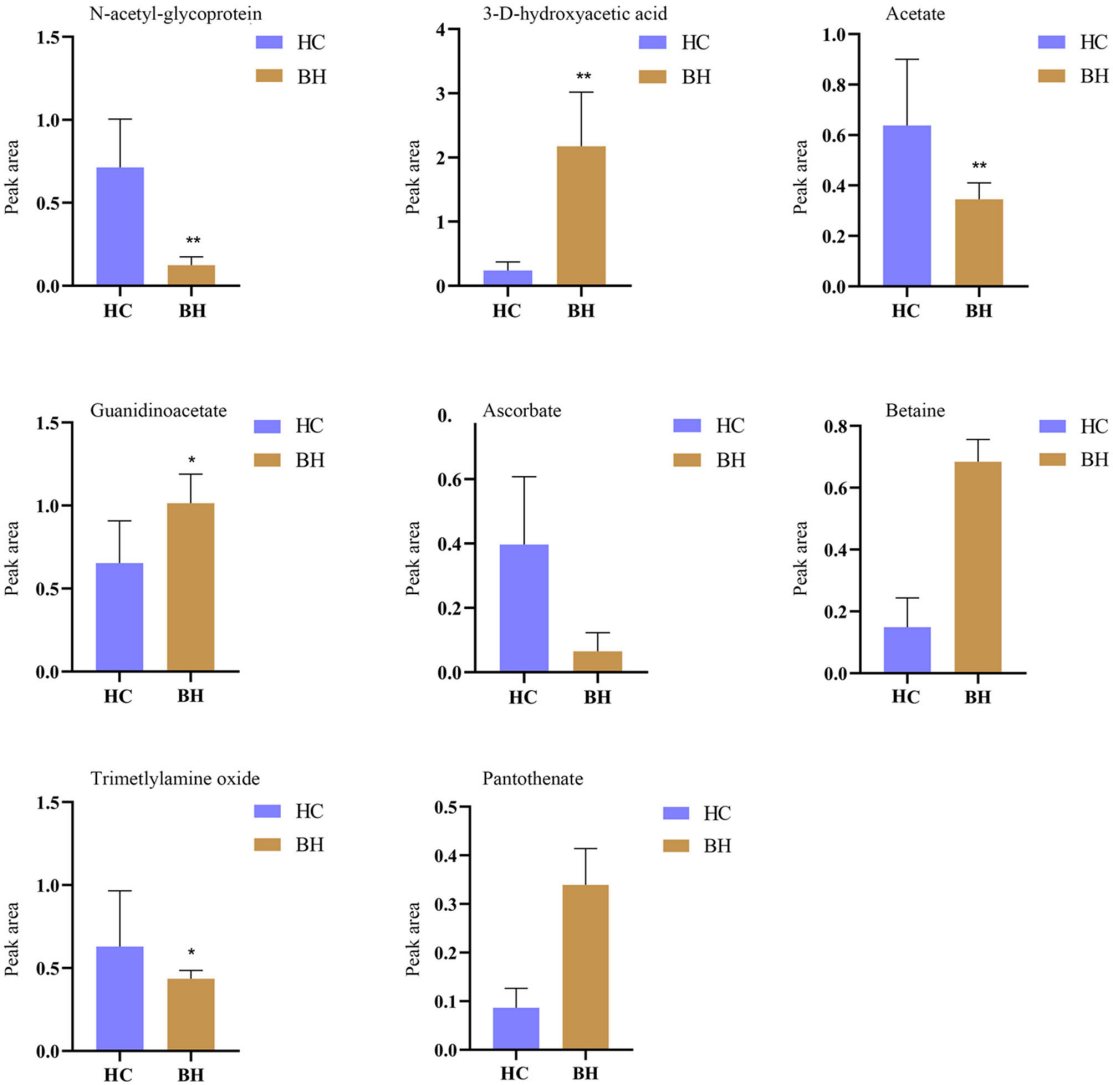
Comparison of relative peak area of potential biomarkers found in serum of BH and HC groups. *P < 0.05, **P < 0.01. BH, bipolar disorder with mania/hypomania episodes; HC, healthy control.

**Figure 8:**
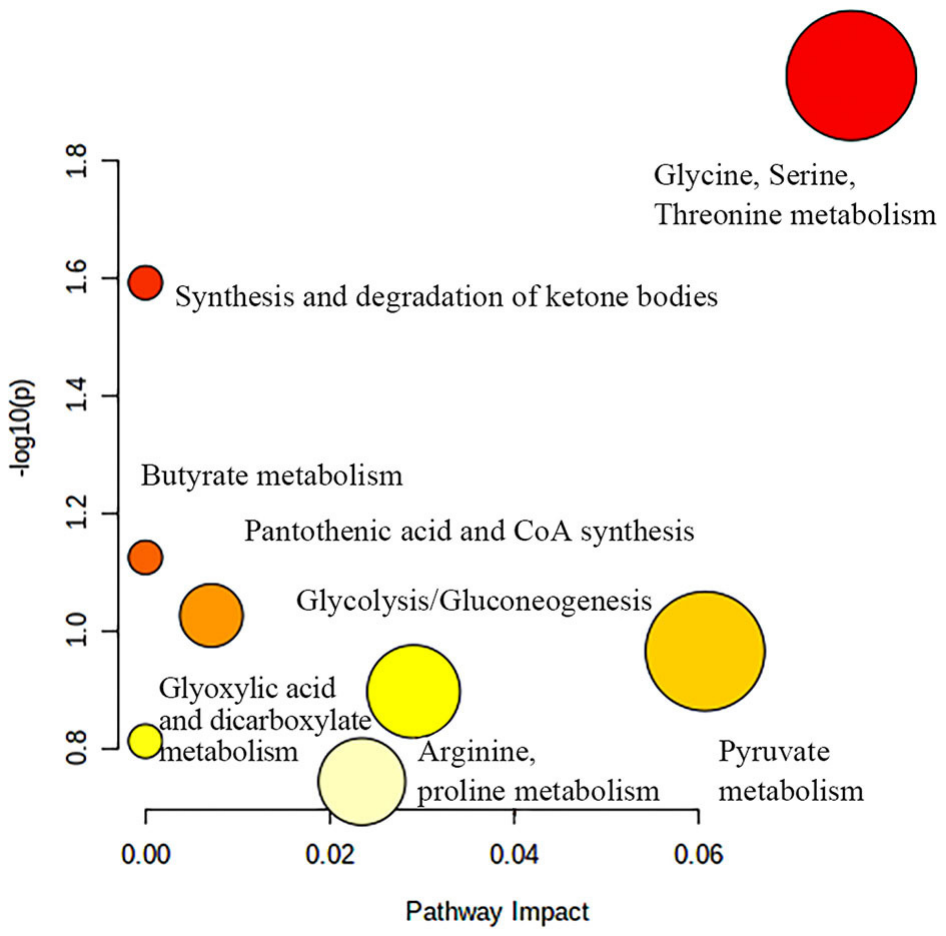
Metabolic pathway analysis of differential metabolites in BH group. BH, bipolar disorder with mania/hypomania episodes.

Figure 7 shows potential biomarkers in the serum of the BH group (Supplementary Table 3). Seven endogenous differential metabolites were identified in the BH group compared to the HC group. Among them, 3-D-hydroxybutyric acid, guanidine acetate, betaine, and pantothenic acid (*P* < 0.05, *P* < 0.01) were significantly elevated compared with those in the HC group. *N*-acetyl-glycoprotein, acetic acid, and oxidized trimethylamine were significantly reduced in the BH group compared with the HC group (*P* < 0.05, *P* < 0.01). The results of the metabolic pathway analysis are shown in Figure 8, which indicate that the model is mainly related to glycine, serine, and threonine metabolism; pyruvate metabolism; glycolysis/glycogenesis; glycerol metabolism; ketone body synthesis and degradation; and arginine and proline metabolism.

**Figure 9:**
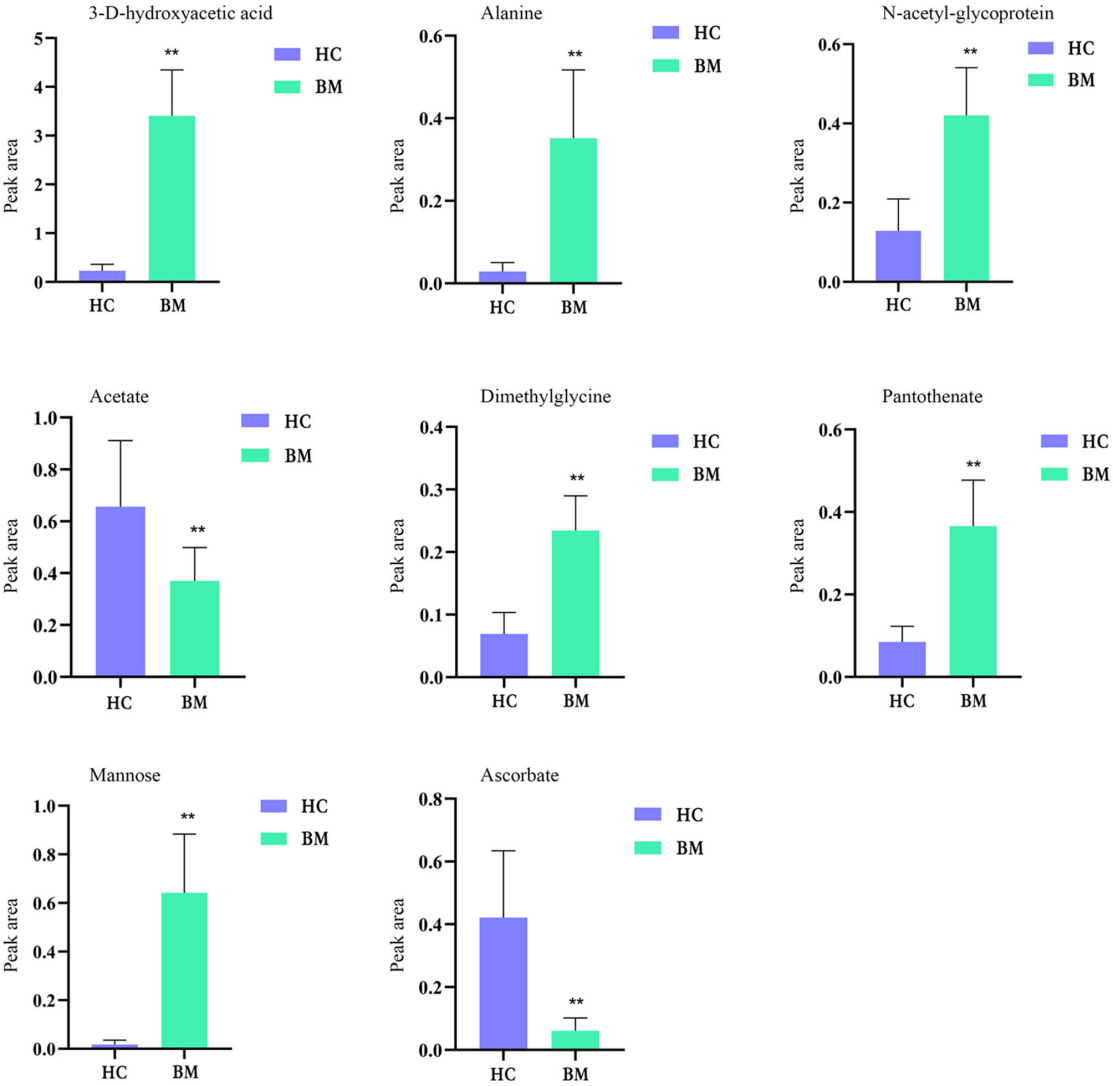
Comparison of relative peak area of potential biomarkers found in serum of BM and HC groups. *P < 0.05, **P < 0.01. BM, bipolar disorder with mixed episodes; HC, healthy control.

**Figure 10:**
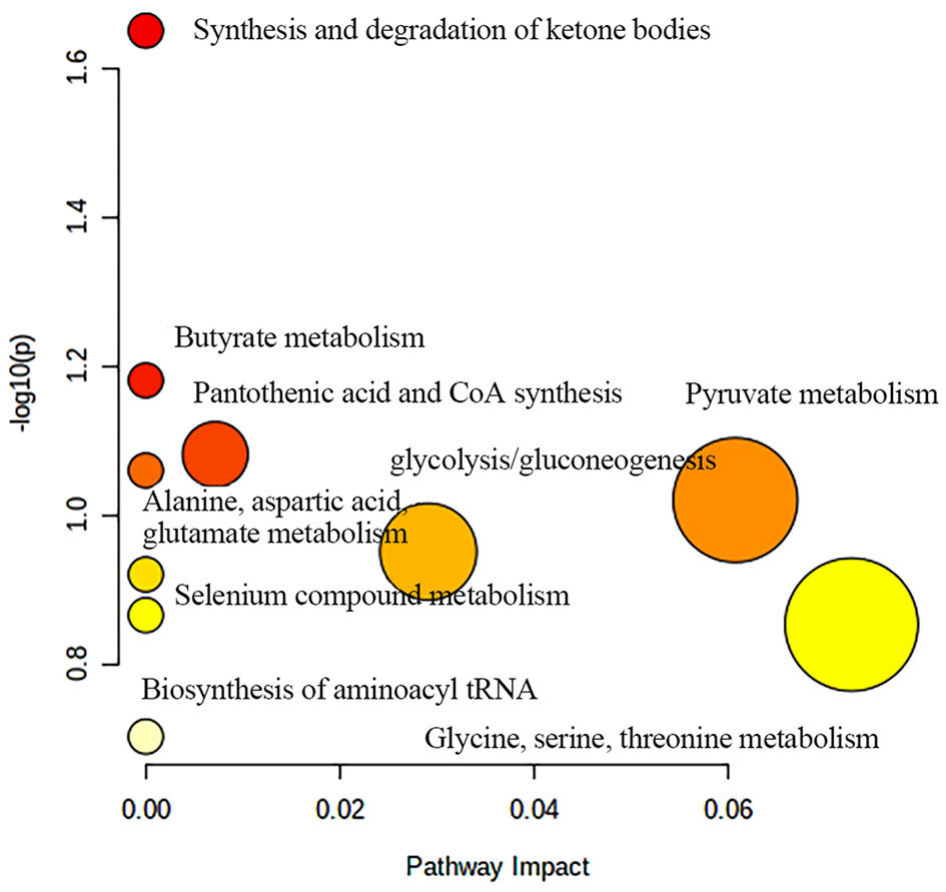
Metabolic pathway analysis of differential metabolites in BM group. BM, bipolar disorder with mixed episodes.

Figure 9 shows the effects of potential biomarkers in the sera of patients with BM (Supplementary Table 4). Compared with the HC group, eight endogenous differential metabolites were identified in the BM group. Among these, 3-D-hydroxybutyric acid, *N*-acetyl-glycoprotein, betaine, pantothenic acid, and mannose levels were elevated compared to those in the HC group (*P* < 0.05), and acetate and ascorbic acids levels were significantly reduced (*P* < 0.05). The model was mainly related to pyruvate metabolism; glycolysis/gluconeogenesis; glycine, serine, and threonine metabolism; ketone body synthesis and degradation; and arginine-proline metabolism (Figure 10).

## 4 Discussion

To the best of our knowledge, this is the first study of the BM metabolic pathway and the metabolomics of BE and BH, providing insights into the diagnosis and pathogenesis of BD. Over the past few years, our research group has been working on this issue and has found that abnormalities in the urea cycle and glutamate and purine metabolisms play important roles in the pathogenesis of non-suicidal self-injury (NSSI)-type BD (18). Another study identified four potential biomarkers for depressive episodes in patients with BD: lactate, oxotrimethylamine, *N*-acetyl glycoprotein, and alpha-glucose (19). In contrast, the present study compared metabolite profiles in participants with different BD clinical phases, focusing on specific metabolic pathways and metabolites in each clinical phase and possible different pathogeneses between clinical phases.

### 4.1 Different BD subtypes share common metabolic pathways and metabolites

Based on the metabolomic analysis results, we found that the three groups of patients with BD had common abnormal metabolic pathways compared with HC, with pyruvate, glycolysis/gluconeogenesis, and ketone body metabolisms predominating in the energy metabolic pathway, which suggests that the BD energy balance may be disturbed, consistent with previous studies (20). Although the cause of elevated pyruvate levels in patients with BD is currently unknown, pyruvate affects mitochondrial adenosine triphosphate (ATP) production because it provides energy to living cells via the citric acid cycle, and growing evidence supports the key role of mitochondrial dysfunction in the pathogenesis of BD (21). Elevated pyruvate levels are likely to play a role in the pathogenesis of BD; however, its exact mechanisms need to be further explored. In addition, we found that ketone body metabolism was the most common metabolic disorder in all three patient groups. One meta-analysis found that oxidative stress plays a role in BD (22), with ketone body metabolism being involved in the inflammatory response and oxidative stress and the possible involvement of ketone body disorders in the pathogenesis of BD. α-Hydroxybutyric acid is present and elevated in all three groups of metabolites in BD, as previously reported (23). Higher concentrations of α-hydroxybutyrate have also been detected in urine, indicating increased oxidative stress in patients with BD (24). Previous studies have also revealed that patients with BE have higher serum N-acetyl-glycoprotein levels than the HC (19), which is consistent with the present study and was found in both BH and BM.

### 4.2 Metabolic pathways and metabolites in BE

The present study found that disturbances in the lipid metabolic pathway during BE affected glycerol metabolism, which is consistent with previous studies (25). Previous studies did not specify the type of episode. In contrast, our study found glycerol metabolism disturbances only in BE and BH and not in the BM, suggesting that glycerol metabolism is only involved in BE and BH. In addition, we found that glycerol, lipids, and acetoacetate metabolites were elevated only in BE and were absent in BH and BM. Previous studies have revealed that lipids, choline, and *N*-acetyl-L-phenylalanine are key metabolites in the serum of patients with BD (26). Moreover, lipid levels are elevated in the brains of patients with BD (27). Based on these findings, glycerol, lipids, and acetoacetate could be used as potential BE markers and provide new diagnostic methods.

### 4.3 Metabolic pathways and metabolites in BH

In the present study, arginine-proline metabolism was abnormal in the BH and BM but not in the BE. Most studies have shown that trimethylamine oxide (TMAO) in the peripheral serum can disrupt and cross the blood-brain barrier (BBB) and act centrally (28), leading to neurobehavioral abnormalities, such as cognitive impairment (29) and depressive conditions (30). Serum TMAO levels are positively correlated with the severity of autism. Cognitive impairment caused by TMAO may be associated with the downregulation of hippocampal methionine sulfoxide reductase expression (31) and increased neuroinflammation and reactive oxygen species production in the hippocampus (32). In addition, TMAO may influence social behavior by regulating metabolites in the hippocampus, which provides new insights into the role of the gut microbiota in regulating social behavior (33). Our study confirmed that TMAO is present only in the BH and is a specific metabolic indicator. TMAO is a harmful and indirect gut flora metabolite (34), and patients with BH frequently have eating disorders. The metabolism of intestinal TMAO is inhibited by the intake of nutrients via microbial enzyme inhibition, and the intake of TMAO-rich foods is controlled to reduce these negative effects. The intervention of the intestinal flora or its metabolites can be indirectly treated by the administration of antibiotics or probiotics, transplantation of flora, or changes in diet. In this study, guanidine acetic acid (GAA) was, for the first time, identified in patients with BD and was present only in mania. Herein, GAA levels significantly increased in BH. Over the last decade, GAA has been mainly studied as a nutritional supplement, and studies have found that dietary GAA may lead to elevated serum homocysteine (35). We hypothesized that GAA provides higher energy in patients with BD with high energy levels and that GAA is only present in BH, implying that GAA is a characteristic bio-criterion for diagnosing BH. Serum GAA levels may be an independent disease-specific biomarker.

### 4.4 Metabolic pathways and metabolites in BM

We also found that some metabolic pathways are uniquely disturbed in patients with BM, including glycine, serine, and threonine metabolism. One study reported that limiting serine and glycine levels in the food of xenograft mice induced the accumulation of deoxysphingosine and reduced tumorigenesis (36). Because serine metabolism regulates giant barred cell polarization, intervention in serine metabolism may be a therapeutic strategy for giant barred cell-mediated immune diseases. Glycogen synthase kinase 3β (GSK-3β) is a serine/threonine protein kinase that mediates the phosphorylation of serine and threonine residues in several target molecules. Compelling evidence that GSK-3 plays a role in the pathophysiology of mood disorders exists, including BD (37). In addition, our study found that acetic and ascorbic acids are metabolites present in BM and BH but are absent in BE. This is the first time that these substances have been identified in patients with BD. Furthermore, acetic and ascorbic acids can be potential biomarkers for BD.

## 5 Conclusion

Although BD various clinical phases share many metabolic pathways, they also have specific metabolic pathways and metabolites. In the present study, we found that TMAO and guanidinoacetic acid metabolites were specific to BH, and glycine, serine, and threonine metabolism was the unique metabolic abnormality in patients with BM. Therefore, these metabolites may be potential biomarkers for BD pathogenesis and contribute to diagnosing and treating different BD. Controlled intake of TMAO-rich foods may correct metabolic disturbances and benefit patients with BD. Future studies should be rigorously designed with larger samples to validate and confirm our results and conclusions. We believe that the biomarkers identified in this study will provide diagnostic support. However, this study has some limitations. First, the sample size was limited, and further studies with larger sample sizes are required. Second, this cross-sectional study focused on the acute phase of the disease and lacked metabolite studies during remission. Moreover, in this study, only serum samples were used; thus, future studies should collect other biological samples, such as cerebrospinal fluid and urine, to ensure that these different sample metabolites are physiologically linked to the pathogenesis of the disease. More studies are required to confirm the role of these metabolites in metabolic pathways.

## Data availability statement

The datasets presented in this study can be found in online repositories. The names of the repository/repositories and accession number(s) can be found in the article/Supplementary material.

## Ethics statement

The studies involving humans were approved by the Ethics Committee of Shanxi Bethune Hospital. The studies were conducted in accordance with the local legislation and institutional requirements. The participants provided their written informed consent to participate in this study.

## Author contributions

QG: Formal analysis, Methodology, Writing – original draft. JJ: Data curation, Formal analysis, Investigation, Supervision, Writing – review & editing. XS: Data curation, Formal analysis, Methodology, Supervision, Validation, Writing – review & editing. HY: Conceptualization, Funding acquisition, Investigation, Project administration, Resources, Software, Supervision, Writing – review & editing. YR: Conceptualization, Data curation, Investigation, Methodology, Project administration, Resources, Software, Supervision, Writing – review & editing.
