# Supplementary material for: Comparing the metabolic pathways of different clinical phases of bipolar disorder through metabolomics studies
Source: Front Psychiatry. 2024 Jan 8;14:1319870. doi: 10.3389/fpsyt.2023.1319870 (PMC10804847; doi:10.3389/fpsyt.2023.1319870)
Supplement: Supplementary file 1 [file Data_Sheet_1.docx]

Supplementary Material

Comparing the metabolic pathways of different clinical subtypes of bipolar disorder through metabolomics studies

Qin Guo*, Jiao Jia, Xiaoli Sun, Hong Yang, Yan Ren

*** Correspondence: renyansxmu@outlook.com**

# Supplementary Figures and Tables

## 1.1 Supplementary Figures


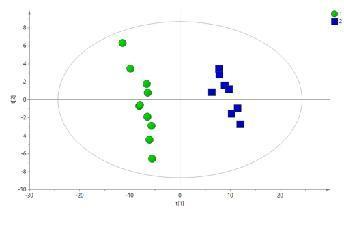

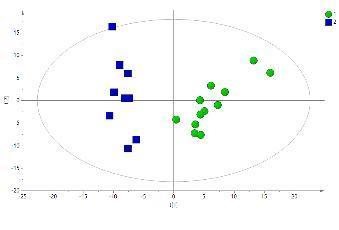

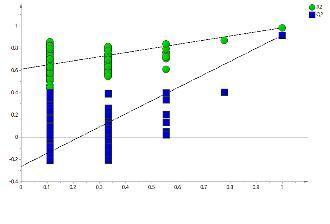
 A B C

**Supplementary Figure 1.** (A) PCA score plots of BH and HC groups. (B) PLS-DA score plots of BH and HC groups. (C) PLS-DA model between BH (circle) and HC (square) groups. Abbreviations: BH, bipolar disorder with mania/hypomania episodes; HC, healthy control; PCA, principal component analysis; PLS-DA, partial least squares discriminant analysis.


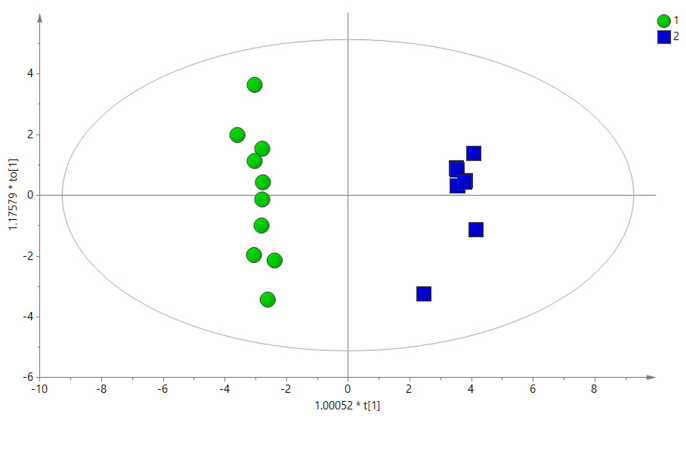

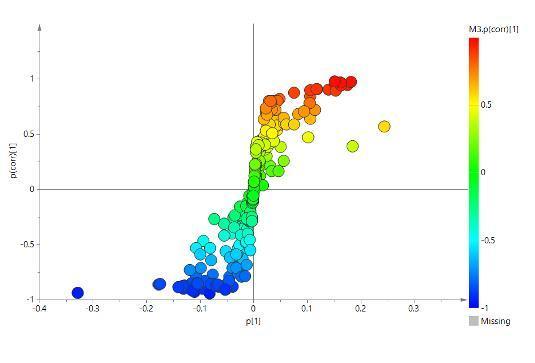


A B

**Supplementary Figure 2.** (A) OPLS-DA score plots of BH and HC groups. (B) PCA plots of the OPLS-DA of BH and HC groups, validated using S-plots. Abbreviations: BH, bipolar disorder with mania/hypomania episodes; HC, healthy control; PCA, principal component analysis; OPLS-DA, orthogonal projections to latent structures discriminant analysis.


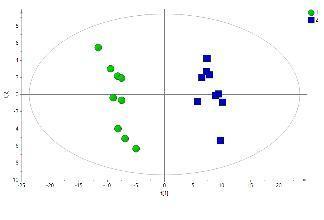

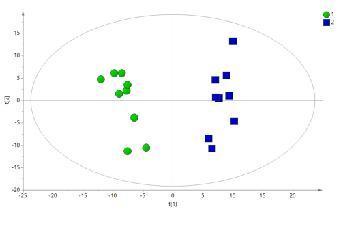

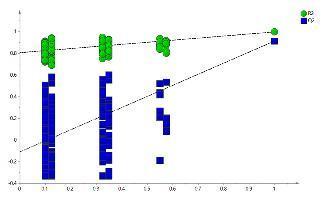


A B C

**Supplementary Figure 3.** (A) PCA score plots of BM and HC groups. (B) PLS-DA score plots of BH and HC groups. (C) PLS-DA model between BM (circle) and HC (square) groups. Abbreviations: BM, bipolar disorder with mixed episodes; HC, healthy control; PCA, principal component analysis; PLS-DA, partial least squares discriminant analysis.


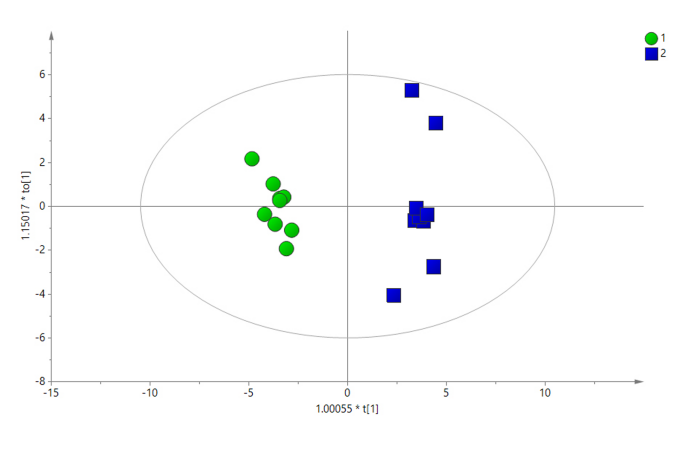

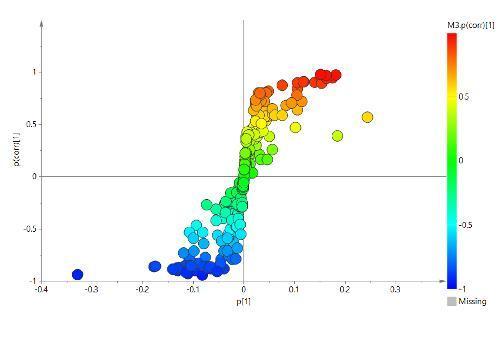


A B

**Supplementary Figure 4.** (A) OPLS-DA score plots of BM and HC groups. (B) PCA plots of the OPLS-DA of BM and HC groups, validated using S-plots. Abbreviations: BM, bipolar disorder with mixed episodes; HC, healthy control; PCA, principal component analysis; OPLS-DA, orthogonal projections to latent structures discriminant analysis.

# 1.2 Supplementary Tables

| **Supplementary Table 1.** Peak attribution in 1H-NMR spectra of differential metabolites. | | |
| --- | --- | --- |
| **No.** | **Metabolites** | **Chemical shift** |
| 1 | Lipids | 0.874（m） |
| 2 | Pantothenate | 0.907（s） |
| 3 | Isoleucine | 0.949（t） |
| 4 | Leucine | 0.961（t） |
| 5 | 3-Hydroxybutyric acid | 1.21（d） |
| 6 | Lactate | 1.33（d） |
| 7 | Acetate | 1.927（s） |
| 8 | O-Acetyl glycoproteins | 2.14（s） |
| 9 | Acetoacetate | 2.28 (s), 3.44 (s) |
| 10 | β-Glucose | 3.25 (dd, 9.4 Hz, 8.1 Hz) |
| 11 | Guanidinoacetate | 3.80 (s) |
| 12 | Pyruvate | 2.37 (s) |
| 13 | Histidine | 7.04 (s), 7.84 (s) |
| 14 | Dimethylglycine | 2.92 (s), 3.70 (s) |
| 15 | Creatine | 3.04 (s), 3.93 (s) |
| 16 | Acetylcholine | 3.23 (s) |
| 17 | Taurine | 3.27 (t, J = 6.6 Hz), 3.42 (t, J = 6.6 Hz) |
| 18 | Mannose | 5.19 (d, 1.6 Hz) |
| 19 | 3-D-hydroxyacetic acid | 1.20 (d) |
| 20 | Betaine | 3.27 (m) |
| 21 | Glyceryl | 3.67 (m), 3.78 (m) |
| 22 | Citrulline | 3.73(s) |
| 23 | N-acetyl-glycoprotein | 2.05 (s) |
| 24 | Glutamate | 2.06 (m), 2.14 (m), 2.36 (m) |
| 25 | Glutamine | 2.14 (m) |
| 26 | Acetone | 2.23 (s) |
| 27 | Citrate | 2.53 (d, 16.1 Hz), 2.70 (d, 16.1 Hz) |
| 28 | Choline | 3.20 (s), 4.06 (m) |

| **Supplementary Table 2.** The peak area of metabolites in serum 1H-NMR spectra of HC group and BE group. | | | |
| --- | --- | --- | --- |
| **Metabolites** | **Peak area after normalization** | |  |
|  | **HC** | **BE** |  |
| 3-D-hydroxyacetic acid | 0.549±1.048 | 2.019±1.272 |  |
| N-acetyl-glycoprotein | 0.185±0.156 | 0.378±0.145 |  |
| β-Glucose | 0.75±0.124 | 0.95±0.211 |  |
| pantothenate | 0.293±0.086 | 0.389±0.092 |  |
| Mannose | 0.069±0.167 | 0.565±0.317 |  |
| Glyceryl | 0.609±0.156 | 0.954±0.273 |  |
| Lactate | 0.428±0.200 | 0.139±0.183 |  |
| Acetoacetate | 0.597±0.164 | 0.346±0.166 |  |
| Lipids | 0.343±0.102 | 0.443±0.114 |  |
| BE, bipolar disorder with depressive episodes; HC, healthy control. |  |  |  |

| **Supplementary Table 3**. The peak area of metabolites in serum 1H-NMR spectra of HC group and BH group. | | | |
| --- | --- | --- | --- |
| **Metabolites** | **Peak area after normalization** | |  |
|  | **HC** | **BH** |  |
| 3-D-hydroxyacetic acid | 0.237±0.135 | 2.173±0.845 |  |
| N-acetyl-glycoprotein | 0.713±0.291 | 0.124±0.051 |  |
| Acetate | 0.668±0.262 | 0.346±0.065 |  |
| Guanidinoacetate | 0.654±0.254 | 1.014±0.174 |  |
| Ascorbate | 0.397±0.211 | 0.065±0.057 |  |
| Betaine | 0.149±0.095 | 0.684±0.071 |  |
| Trimetlylamine oxide | 0.630±0.337 | 0.435±0.051 |  |
| pantothenate | 0.087±0.039* | 0.340±0.074 |  |
| BH, bipolar disorder with mania/hypomania episodes; HC, healthy control. |  |  |  |

| **Supplementary Table 4**. The peak area of metabolites in serum 1H-NMR spectra of HC group and BM group. | | | |
| --- | --- | --- | --- |
| **Metabolites** | **Peak area after normalization** | |  |
|  | **HC** | **BM** |  |
| 3-D-hydroxyacetic acid | 0.226±0.133 | 3.407±0.111 |  |
| N-acetyl-glycoprotein | 0.129±0.081 | 0.420±0.121 |  |
| Alanine | 0.029±0.021 | 0.352±0.165 |  |
| Acetate | 0.656±0.255 | 0.371±0.128 |  |
| Dimethylglycine | 0.069±0.034 | 0.234±0.055 |  |
| pantothenate | 0.084±0.038 | 0.365±0.111 |  |
| Mannose | 0.017±0.018 | 0.642±0.041 |  |
| Ascorbate | 0.421±0.213 | 0.061±0.041 |  |
| BM, bipolar disorder with mixed episodes; HC, healthy control. |  |  |  |
